# Baricitinib treatment for refractory skin changes in POEMS syndrome: a case report

**DOI:** 10.3389/fphar.2023.1191158

**Published:** 2023-09-12

**Authors:** Jingjing Xie, Zhiling Li, Yubao Jiang, Dabin Tang, Xia Qiu, Ertao Jia, Jianyong Zhang

**Affiliations:** ^1^ The Fourth Clinical Medical College of Guangzhou University of Chinese Medicine, Shenzhen, Guangdong, China; ^2^ The Department of Rheumatology, Shenzhen Traditional Chinese Medicine Hospital, Shenzhen, Guangdong, China; ^3^ Guangzhou University of Chinese Medicine, Guangzhou, China

**Keywords:** POEMS syndrome, Janus kinase inhibitor (JAKI), JAK/STAT signaling, skin changes, case report

## Abstract

Polyneuropathy, organomegaly, endocrinopathy, monoclonal plasma cell disorder, and skin changes (POEMS) syndrome is a multisystem disorder that has limited treatment options. Here, we described a case of a 55-year-old female subject who was treated for multiple drugs, but the skin symptoms continued to progress; the patient responded well to baricitinib. This suggests that JAK/STAT signaling pathways play an essential role in the pathological process of POEMS syndrome.

## 1 Introduction

Polyneuropathy, organomegaly, endocrinopathy, monoclonal plasma cell disorder, and skin changes (POEMS) syndrome is a multisystem disorder that has limited treatment options (Brown and Ginsberg, 2019). Majority of patients with POEMS syndrome present with hyperpigmentation, hemangiomas, and hypertrichosis. However, most patients do not benefit from the current approaches available for the treatment of these cutaneous symptoms. Herein, we report the case of a patient with POEMS syndrome who presented with six types of skin changes and responded well to baricitinib, with all skin lesions resolving over the treatment period.

## 2 Case report

The patient is a 55-year-old female subject, who, in 2014, was initially diagnosed with systemic sclerosis due to pulmonary fibrosis and skin changes, including hypertrichosis of the limbs and jaws, thickening and swelling of the skin, hyperpigmentation, hemangiomas, Raynaud’s phenomenon, and swelling of the back and lower limbs. Skin biopsy of the right leg revealed hyperkeratosis of the epidermis, mild hyperplasia of the dermis, and sparse collagen fibers in the middle without dermal vascularization. In 2016, the patient complained of bilateral lower limb pain, numbness, and paresthesia. An electrophysiological examination suggested multiple peripheral neuropathies. Ultrasonography revealed bilateral lymphadenopathy in the neck and supraclavicular, axillary, inguinal, mesenteric, and retroperitoneal regions. Laboratory tests revealed diabetes mellitus and mild renal dysfunction, as well as serum vascular endothelial growth factor (VEGF) levels of 7,924 pg/mL (normal range: 0–600 pg/mL). Furthermore, serum protein electrophoresis revealed a monoclonal band of IgA lambda +. Consequently, the patient was diagnosed with POEMS syndrome due to multiple peripheral neuropathy symptoms, skin changes, and a high level of VEGF, based on the current diagnostic criteria for POEMS syndrome, i.e., the Dispenzieri diagnostic criteria (Miest et al., 2013).

**FIGURE 1:**
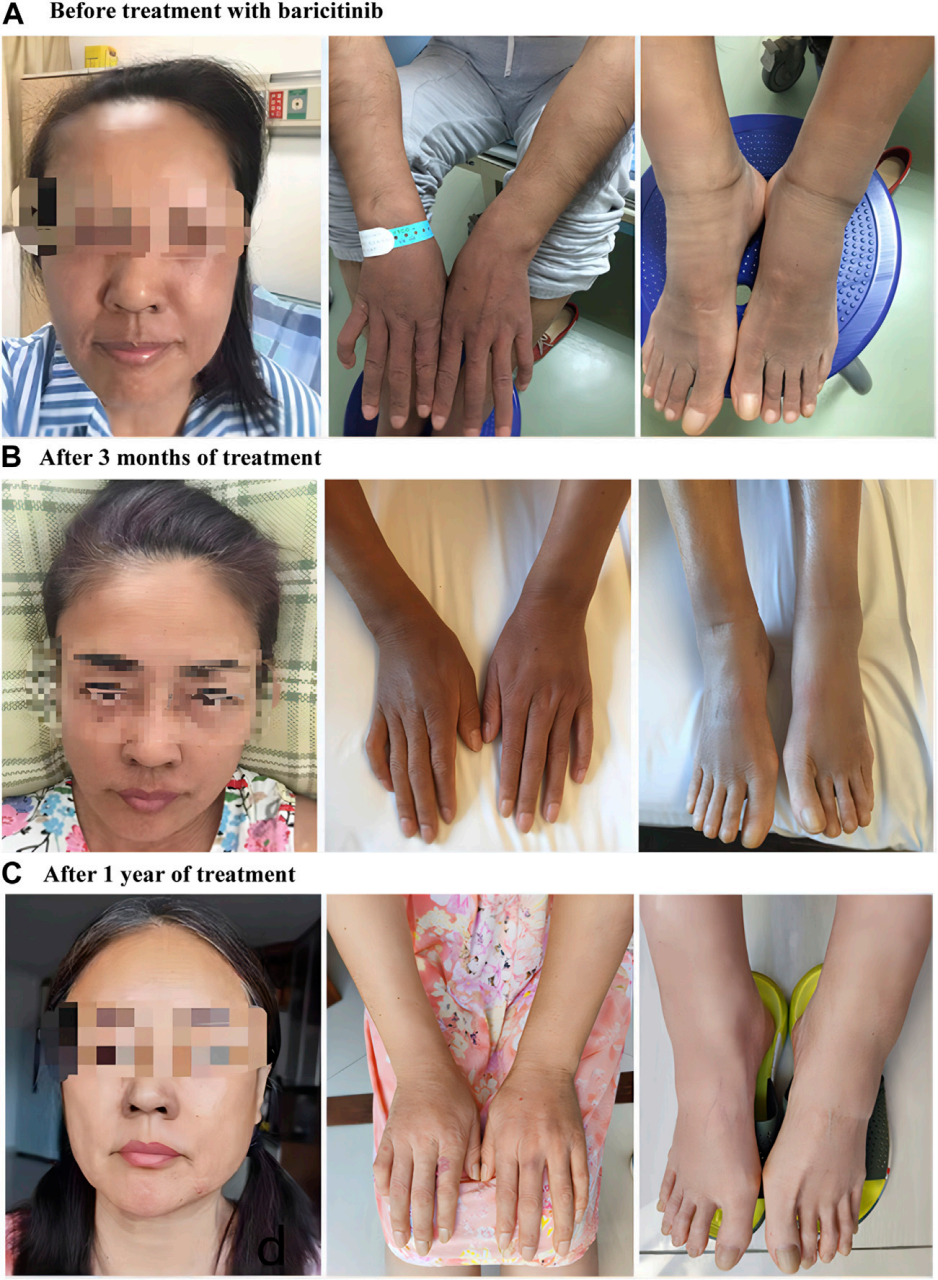
Prior to the treatment with baricitinib, hyperpigmentation, skin thickening on the face, upper and lower limbs with hypertrichosis and hemangiomas in the extensor lateral forearms, and swollen lower limbs were observed **(A)**. The skin changes have partially dissipated after 3 months of treatment **(B)** and have been almost fully reversed after 1 year **(C)**.

The patient refused bortezomib; therefore, an alternative treatment regimen was adopted. Despite treatment with multiple drugs, including thalidomide, cyclophosphamide, glucocorticoids, and Kunxian capsules, supplemented with the usual dose, the skin symptoms continued to progress, while other non-skin symptoms improved. Hence, the treatment plan was modified, and oral baricitinib (2 mg/day) was administered. The cutaneous symptoms improved after 3 months of baricitinib treatment, and full recovery was noted after 1 year (Figure 1). The patient showed sustained improvement in the skin symptoms over 18 months of follow-up, and serum VEGF levels decreased considerably to 50.62 pg/mL.

## 3 Discussion

The Janus kinase (JAK)/STAT pathway is involved in various skin disorders, including alopecia areata, atopic dermatitis, lupus erythematosus, dermatomyositis, psoriasis, and vitiligo (Solimani et al., 2019). Vitiligo is an autoimmune skin disease in which melanocytes are reduced in association with the activation of the interferon-γ (IFN-γ) and CXCL-10 signaling pathways (Frisoli et al., 2020). The JAK inhibitor baricitinib, which has been shown to block IFN-γ signaling and contribute to re-pigmentation, has been approved for treatment of patients with vitiligo (Qi et al., 2021; Yan et al., 2022). Interestingly, in the present case, hyperpigmentation was treated with baricitinib, which is in contrast to the skin symptoms of vitiligo.

To the best of our knowledge, this is the first report on the successful use of baricitinib to treat skin symptoms associated with POEMS syndrome. The pathogenic mechanisms underlying the skin symptoms associated with POEMS syndrome are poorly understood. However, VEGF has been identified as a key cytokine involved in the pathogenesis of this disorder and is known to reflect the disease activity (Dispenzieri, 2017). Du et al. reported that VEGF expression is associated with JAK/STAT signaling in psoriasis (Du et al., 2020). The role of JAK/STAT in rheumatoid arthritis and new vessel formation has also been reported. Paola et al. explored the anti-angiogenic role of tofacitinib, another JAK inhibitor, in experimental arthritis, which was attributed to inhibition of the pro-angiogenic effects of VEGF (Di Benedetto et al., 2021). Further evidence has confirmed that among the tyrosine kinase cell receptor (VEGFR) 2-mediated signaling pathways, the JAK/STAT signaling pathway, especially the STAT3 signaling pathway, is a critical target and biomarker of angiogenesis (Zhang et al., 2011). Given these findings and our observation with the present case, we propose that angiogenesis is the primary process involved in the pathogenesis of cutaneous changes in patients with POEMS syndrome.

## 4 Conclusion

Baricitinib is a promising treatment for skin symptoms in patients with POEMS, which can be attributed to the targeting of JAK/STAT signaling to inhibit angiogenesis. However, further studies are required to better understand the pathogenic mechanisms underlying POEMS syndrome, specifically the role of JAK/STAT3 signaling and angiogenesis.

## Data Availability

The original contributions presented in the study are included in the article/Supplementary material; further inquiries can be directed to the corresponding authors.
